# Advances of 3D printing technologies in orthopaedic trauma and surgical training: a transformative approach

**DOI:** 10.1007/s00068-024-02464-4

**Published:** 2024-02-14

**Authors:** Jonas Neijhoft, Frank FA IJpma²

**Affiliations:** 1 Department of Trauma, Hand and Reconstructive Surgery, Goethe University Frankfurt, Theodor- Stern-Kai 7, 60590 Frankfurt, Germany; 2 Department of Trauma Surgery, University of Groningen, University Medical Center Groningen, Groningen, The Netherlands

The field of trauma surgery, orthopaedics, and surgical training has witnessed a paradigm shift with the advent of three-dimensional (3D) printing technologies. Although 3D printing is not a completely new achievement, the areas of application have been increasingly integrated into everyday clinical practice in recent years. In trauma surgery and orthopaedics in particular, the potential applications are very diverse and promising. 3D technologies can be used across various stages of a patient’s medical journey. Virtual and printed 3D models might serve as valuable educational tools for medical students, residents, and surgeons, aiding in enhancing their understanding of injuries [1, 2]. In the preoperative phase, surgeons can employ virtual 3D fracture models for surgical planning, facilitating the analysis of complex fracture patterns and optimizing the placement of implants [3]. During surgery, 3D technology can offer real-time guidance to implement the preoperative plan into the actual operation. Surgical guides produced through 3D printing can be directly placed in the surgical field, aiding in osteotomy saw planes, fracture reductions, and the positioning of implants and screws [4]. Postoperatively, 3D imaging enables the assessment of surgical outcomes concerning the quality of fracture reduction and the accuracy of implant placement [5]. 

This special issue compiles five papers that delve into the applications, potential benefits, and challenges of 3D technologies in various aspects of orthopaedic trauma surgery.

Jörgens et al. [6] explore the realm of precision in high tibial osteotomy (HTO) using 3D-printed patient-specific instruments. The study evaluates the approach of 3D-printed-assisted HTO regarding its accuracy in comparison to traditional methods. The results indicate that 3D cutting blocks and spacers facilitate the translation of 3D planning into reality with minimal deviations, highlighting the potential of this method in patient specific HTOs.

The second paper delves into patient-specific osteosynthesis for medial tibial plateau fractures, introducing a workflow that aims to facilitate proper fracture reduction. Assink et al. [7] showcase the feasibility of a patient-specific approach, demonstrating improved outcomes in terms of articular reduction, plate positioning, and screw direction. 3D surgical planning was performed, and a patient-specific implant was designed and produced for fracture fixation. This research lays the foundation for a personalized and precise approach to tibial plateau fracture surgery.

A comprehensive overview regarding the impact of 3D printing-assisted pre-operative planning on foot and ankle fracture fixation is provided by Wood et al. [8] Through a systematic review and meta-analysis, the study demonstrates significant improvements in operation duration, intraoperative blood loss, fluoroscopy usage, and overall ankle health. The findings provide promising evidence for the use of 3D printing in enhancing surgical outcomes for foot and ankle fractures.

Oldhoff et al. [9] address the ongoing debate regarding type of implants used for performing corrective osteotomies of the distal radius. By comparing 3D-assisted correction osteotomy with use of pre-contoured conventional implants versus patient-specific implants, the study contributes valuable insights. The findings reveal that both approaches result in accurate corrections, emphasizing that the choice of implant type should consider factors beyond accuracy, such as resource availability and preoperative implant fitting assessment.

A last paper focuses on the educational aspect of 3D printing in early education of trauma surgery [10]. It evaluates the effectiveness of 3D-printed hands-on radius fracture models in enhancing medical students’ understanding of fracture anatomy. The results indicate that the 3D models, when combined with traditional imaging methods, improve students’ ability to classify fractures, highlighting the potential of 3D visualization in medical education.

In conclusion, these papers collectively underscore the transformative potential of 3D printing in trauma surgery, orthopaedics, and surgical training. As the field continues to evolve, the integration of 3D technologies promises to revolutionize surgical practices, improve outcomes, and enhance medical education. This special issue contributes to the growing body of knowledge, paving the way for future innovations and advancements in the use of 3D technologies in orthopaedic trauma surgery.
